# A Novel Small Molecule, 1,3-di-m-tolyl-urea, Inhibits and Disrupts Multispecies Oral Biofilms

**DOI:** 10.3390/microorganisms8091261

**Published:** 2020-08-20

**Authors:** Shanthini Kalimuthu, Becky P.K. Cheung, Joyce Y.Y. Yau, Karthi Shanmugam, Adline Princy Solomon, Prasanna Neelakantan

**Affiliations:** 1 Faculty of Dentistry, The University of Hong Kong, Pok Fu Lam, Hong Kong; shanthini.ks1609@gmail.com (S.K.); bpkcheun@hku.hk (B.P.K.C.); yaujyy@hku.hk (J.Y.Y.Y.); 2 Quorum Sensing Laboratory, Center of Research in Infectious Diseases, School of Chemical and Biotechnology, SASTRA Deemed to be University, Thanjavur 613401, India; karthi.bioinfo@gmail.com

**Keywords:** DMTU, multispecies biofilms, *Porphyromonas gingivalis*, quorum sensing

## Abstract

An imbalance of homeostasis between the microbial communities and the host system leads to dysbiosis in oral micro flora. DMTU (1,3-di-m-tolyl-urea) is a biocompatible compound that was shown to inhibit *Streptococcus mutans* biofilm by inhibiting its communication system (quorum sensing). Here, we hypothesized that DMTU is able to inhibit multispecies biofilms. We developed a multispecies oral biofilm model, comprising an early colonizer *Streptococcus gordonii*, a bridge colonizer *Fusobacterium nucleatum*, and late colonizers *Porphyromonas gingivalis* and *Aggregatibacter actinomycetemcomitans.* We performed comprehensive investigations to demonstrate the effect of DMTU on planktonic cells and biofilms. Our findings showed that DMTU inhibits and disrupts multispecies biofilms without bactericidal effects. Mechanistic studies revealed a significant down regulation of biofilm and virulence-related genes in *P. gingivalis*. Taken together, our study highlights the potential of DMTU to inhibit polymicrobial biofilm communities and their virulence.

## 1. Introduction

Microbial communities exist in homeostasis with the host in healthy individuals. However, factors including epigenetic and genetic changes, and stress conditions such as smoking and systemic diseases, trigger an imbalance in this homeostasis by creating dysbiosis within microbial communities [1,2]. Such dysbiosis of the oral microflora is responsible for several costly diseases, including dental caries, periodontitis and peri-implant infections [3,4]. The keystone pathogen in the periodontal and peri-implant niches is *Porphyromonas gingivalis* [5,6]. This Gram-negative, obligate anaerobe is a late colonizer. It is non-motile, asaccharolytic, and requires hemin and vitamin K in its milieu [2,7]. Since this pathobiont usually resides in deep periodontal pockets, characterized by carbohydrate limitation, it procures energy by amino acid fermentation [8,9]. *P. gingivalis* plays an important role in tissue breakdown by modulating the host immune response and invading epithelial cells via the production of several proteases (gingipains) [10]. This results in the release of collagen and heme, which are used as nutritional sources for further growth and biofilm development [1,11]. There are several other mechanisms by which *P. gingivalis* modulates and escapes from the host immune system. For instance, *P. gingivalis* fimbriae, gingipains and lipopolysaccharides (LPS) prevent leukocyte recruitment to the diseased site by degrading the macrophage receptor CD14, and invade the epithelial cells, which then upregulates the expression of E-selectin and prevents leukocyte adhesion [12]. Macrophage polarization M1/M2 is maintained as a response to chronic inflammatory responses, but studies have shown that patients with periodontitis show reduced macrophage polarization, thus eventually affecting the host immune surveillance [13]. However, animal studies have demonstrated that *P. gingivalis* alone is incapable of causing virulence in germ-free mice [14]. Collectively, these findings suggest that oral dysbiosis, due to the interplay and cross-talk between *P. gingivalis* and other oral organisms, plays a significant role in modulating the host immune response [12,13].

Different species in the vast oral flora share metabolic pathways, co-exist synergistically and construct themselves to form a highly spatio-temporally organized community, which is referred to as the “polymicrobial synergy and dysbiosis” model (PSD) [15]. In such polymicrobial communities, other bacterial species may impact the growth and metabolism of *P. gingivalis*. For instance, *Streptococcus gordonii*, which is generally non-pathogenic, may initiate colonization and provide metabolic support to *Porphyromonas gingivalis* [16]. Some bacterial species, such as Streptococcus and Actinomyces, are defined as accessory pathogens due to their ability to adhere to the surface of teeth via the salivary pellicle [17,18]. *Fusobacterium nucleatum*, which possess multiple adhesins, is a “bridge colonizer”, since it can attract and attach to several late colonizers, including *P. gingivalis* and *Aggregatibacter actinomycetemcomitans* [16], forming a highly virulent biofilm. Therefore, *S. gordonii, P. gingivalis, F. nucleatum* and *A. actinomycetemcomitans* exhibit excellent synergistic interactions, forming a well-organized biofilm community [19,20]. Overall, the virulence and the pathogenic potential of biofilms is a collective result of inter-species communication and host–microbe interactions [15,21,22].

The central dogma of such polymicrobial communities is that these bacteria exhibit varying phenotypic expression as opposed to monocultures, enhancing the virulence and persistence in host cells [23]. *P. gingivalis* interacts with the early colonizer *S. gordonii* through receptor–adhesin interactions. Once this interaction is initiated, it leads to the dephosphorylation of tyrosine kinase, *Ptk1* which then suppress the expression of *luxS* suppressor gene, *cdrR.* Thus, the LuxS/Autoinducer-2 (AI-2) signaling, which is responsible for inter-species community development, is initiated [24]. Furthermore, it has been demonstrated that AI-2 is required for biofilm development by *A. actinomycetemcomitans* and the activation of adhesion-related genes in *F. nucleatum* [24,25]. Therefore, the LuxS system plays a critical role in the synergistic metabolic relationship amongst the microbes in a polymicrobial community.

As antibiotic resistance strains are emerging worldwide, there has emerged an effort towards the development of new antimicrobials [26]. Although chlorhexidine (CHX) remains the gold standard in oral antiseptics, its indiscriminate broad-spectrum microbicidal effects and long-term exposure lead to the development of resistance in various pathogens. Furthermore, it has limited effects on biofilms [27,28]. Therefore, a hot topic in contemporary microbiological research is the identifying of treatments that can control biofilm dysbiosis and microbial virulence without microbicidal effects. Some of these include probiotics that enhance the growth and development of commensal microbes [29,30], and the developing of small molecules [31] and natural compounds [32] that target the virulence pathways of the bacteria and fungi without affecting microbial growth.

DMTU (1,3-di-m-tolyl-urea) is a biocompatible, aromatic compound which has been reported to demonstrate antibiofilm effects against the cariogenic bacterium, *Streptococcus mutans*, by inhibiting its quorum sensing pathway (comDE). Notably, it has immunomodulatory and anti-infective properties in Wistar rats infected with *S. mutans* [33,34]. Other urea derivatives, such as (S-3, 4-dicholoro benzene)-isothiourea hydrochloride, have been shown to competitively binds to mreB, a cell wall protein that is widely present in Gram-negative bacteria. Thereby, it alters the cell shape affects the adhesion, biofilm formation and motility of *Pseudomonas aeruginosa* without any toxic effects on human cells [35,36]. Thus, urea derivatives appear to be effective antibiofilm agents against both Gram-positive and Gram-negative bacterial pathogens. However, the effect of DMTU on polymicrobial biofilms remains to be investigated. Here, we asked if DMTU is able to inhibit the formation of multispecies biofilms and disrupt preformed biofilms. Our results revealed that DMTU inhibits multispecies biofilm development and disrupts preformed biofilms without any effect on bacterial viability.

## 2. Materials and Methods

### 2.1. Chemicals, Bacterial Strains, and Culture Conditions

*Porphyromonas gingivalis* ATCC 33277, *Fusobacterium nucleatum* CCUG 9126, *Aggregatibacter actinomycetemcomitans* ATCC 33384 and *Streptococcus gordonii* ATCC 35105 were acquired from the American Type Culture Collection (ATCC). The cultures were maintained in Horse Blood Agar (HBA) supplemented with 5 µg/mL of Hemin and 1.0 µg/mL of Vitamin K at 37 °C in an anaerobic chamber (5% CO_2_, 10% H_2_ and 85% N_2_). For all the experiments, the bacteria were grown for 72 h in Tryptic Soy Broth (TSB) supplemented with 5 g/L of Yeast extract (YE), 5 µg/mL of hemin and 1.0 µg/mL of vitamin K at 37 °C [37] in an anaerobic chamber (5% CO_2_, 10% H_2_ and 85% N_2_).

DMTU stock was prepared using 1% DMSO (Sigma Aldrich, St. Louis, MO., United states) as a solvent [33]. In all the experiments, growth media without DMTU served as the positive control while media with 1% DMSO was considered as a vehicle control. Growth medium without the culture served as a negative control. All experiments were performed in triplicates in three independent experiments.

### 2.2. Effect of DMTU on Bacterial Growth

The effect of DMTU on the growth of each bacterial species in monoculture was assessed by using the broth microdilution assay [38]. Individual bacterial suspensions were prepared as mentioned above. Then, DMTU was serially diluted two-fold in media (TSB + YE + Hemin + Vitamin K) to achieve concentrations ranging from 400 µM to 1.6 µM. The microbial suspension was added into the wells of 96-well polystyrene plates and incubated for 24 h at 37 °C in an anaerobic chamber. Appropriate controls were included as mentioned above. After incubation, bacterial growth inhibition was evaluated by measuring the OD at 660 nm using a SpectraMax 340 tunable microplate reader (Molecular Devices, San Jose, CA, USA).

### 2.3. Effects of DMTU on Inhibition of Biofilms

#### 2.3.1. Co-Culture and Biofilm Formation

For establishing biofilms, 72 h grown individual bacterial suspensions were centrifuged at 14,000× *g* for 10 min and the pellet was resuspended and washed twice with PBS to remove the dead cells. For each bacterial suspension, the inoculum was standardized to OD_660_ of 0.271–0.279 to obtain a final concentration of 2 × 10^8^ CFU/mL. For establishing multispecies biofilms, the bacterial suspensions were co-cultured in the ratio of 1:1:1:1 in sterile 96-well microtiter plates for 24 h in an anaerobic chamber [39].

#### 2.3.2. Biofilm Inhibition by Sub-Inhibitory Concentrations of DMTU

The potential effect of sub-inhibitory concentrations (Sub-MIC) of DMTU on biofilm inhibition was investigated by quantifying the biofilm mass using the safranin assay [40,41]. Briefly, biofilms were developed for 24 h to allow initial adhesion and then incubated with DMTU for 24 h at 37 °C in an anaerobic chamber. Then, the planktonic cells were removed and the wells were washed twice with PBS to remove the non-adherent/dead cells. The biofilms were then stained with 0.1% safranin and incubated for 20 min at room temperature. After incubation, the excess stain was removed by washing twice with PBS and plates were dried for 30 min. The stain was then dissolved using 33% acetic acid and the biofilm biomass was quantified by measuring the absorbance at 492 nm.

### 2.4. Effect of DMTU on P. gingivalis Specific Genes

To elucidate the effect of DMTU on biofilm- and virulence-related genes of *P. gingivalis*, quantitative Real Time PCR (qRT-PCR) was performed. Multispecies biofilms were developed in the presence of a biofilm inhibitory concentration of DMTU (0.79 µM) as mentioned above. The planktonic cells were removed by washing twice with PBS. The biofilms were then scraped and centrifuged at 14,000× *g* for 10 min. Total RNA was extracted from the pellet as per the manufacturer’s instructions using the Promega SV total RNA isolation Kit (Promega, Madison, WI, USA). Using Nanodrop, the purity and concentration of RNA were determined. RNA was reverse transcribed to cDNA using High-Capacity cDNA Reverse Transcription kits (Applied Biosystems, Foster City, CA, USA).

The sequence of primers used in this study is listed in Table S1. Each PCR reaction was performed with a total reaction volume of 20 µL containing 10 µL of SYBR green master mix, 1 µL each of forward and reverse primers, 1 µL of diluted cDNA and 4 µL of nuclease-free water. 16S rRNA was used as a house-keeping gene and to calculate the relative changes in gene expression. Gene expression changes were calculated using the 2^−ΔΔ*C*t^ method and expressed as a reduction in relative fold change compared to the control.

### 2.5. Effects of DMTU on Preformed Biofilms

#### Quantification of Biomass and Cell Viability

The effect of DMTU on the biomass and cell viability of established biofilms was quantified using the safranin and XTT assays, respectively. A mature biofilm was established by co-culturing the bacteria for 48 h at 37 °C in an anaerobic chamber. Then, the planktonic cells were removed by washing twice with PBS. Varying concentrations of DMTU (1.56 µM–62.5 µM) were added to the biofilm and the plates were incubated at 37 °C for 24 h in an anaerobic chamber. A safranin assay was performed to quantify the biomass as mentioned above.

To assess the viability of the bacterial cells in DMTU-treated biofilms, an XTT assay was performed [42]. XTT solution (1 mg/mL) was prepared freshly with menadione in PBS at a ratio of 79:20:1 (PBS: XTT: menadione). The planktonic cells were removed, the biofilms were washed carefully with PBS, and 200 µL of XTT solution was added and it was incubated for 3 h in dark conditions at 37 °C. The plates were then centrifuged at 3000 rpm for 5 min. The supernatant was then carefully transferred to new 96-well plates and the absorbance was read at 492 nm.

### 2.6. Confocal Laser Scanning Microscopic Analysis of the Effect of DMTU on Biofilms

#### 2.6.1. Biofilm Inhibition on DMTU-Coated Substrates

The biofilm inhibitory concentrations of DMTU (0.79 µM, 1.56 µM, 3.15 µM) were coated on cover slips in chamber slides (idibi, Fitchburg, WI., USA) and allowed to dry overnight at 37 °C. The bacterial suspensions were then inoculated and incubated for 48 h in an anaerobic chamber at 37 °C. Following incubation, the biofilms were gently washed with PBS and stained using the Live/Dead stain (BacLight Viability kit, Thermo Scientific, Waltham, MA, USA). Biofilm z-stacks were obtained from 5 different spots using a confocal laser scanning microscope (Fluoview FV2000, Olympus, Tokyo, Japan). The total attached bacterial cells/mm^2^ was quantified using the cell-C software [43].

#### 2.6.2. Effect of DMTU on Preformed Biofilms

To visualize the effect of DMTU on preformed biofilms, the treated biofilms were stained with a SyPRO biofilm matrix stain and the bacterial cells were counterstained with Syto9. Briefly, the biofilms were developed for 48 h and then treated for 24 h with different concentrations of DMTU. Following incubation, the planktonic cells were removed and the biofilm was washed twice with PBS. The biofilms were then stained and z-stacks were obtained from 5 different spots using a confocal laser scanning microscope, and the images were processed as mentioned above.

### 2.7. Statistical Analysis

All the assays were carried out in triplicates for three independent trials and the results were expressed as mean ± SD. Statistical analysis of the data was performed by one-way ANOVA (GraphPad Prism version 6.05). *p* ≤ 0.05 was considered statistically significant.

## 3. Results and Discussion

### 3.1. DMTU Inhibits Multispecies Biofilms without Affecting Bacterial Growth

**Figure 1:**
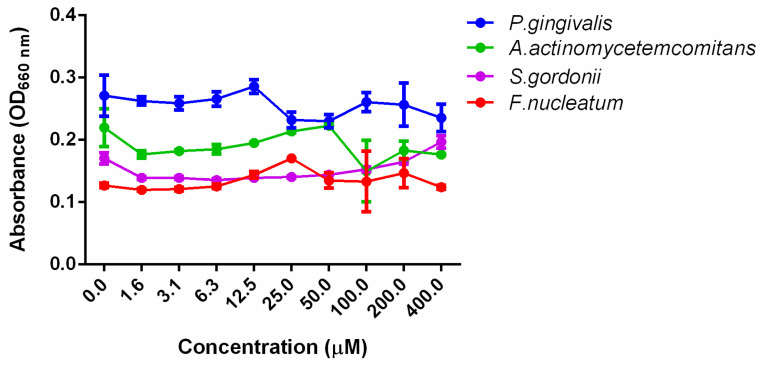
Effect of DMTU on planktonic cells. DMTU did not significantly inhibit bacterial growth up to 400 µM.

Oral biofilms infections are attributed to biofilm dysbiosis. Indiscriminate microbial killing has the potential to result in the development of antimicrobial resistance due to the activation of efflux pumps and the modification of the drug target binding site [44]. Therefore, we aimed at developing a molecule that targets biofilms and virulence without affecting growth. Our results showed that DMTU, in the tested range of concentrations, did not affect the growth of *P. gingivalis, F. nucleatum, S. gordonii* or *A. actinomycetemcomitans* (Figure 1).

**Figure 2:**
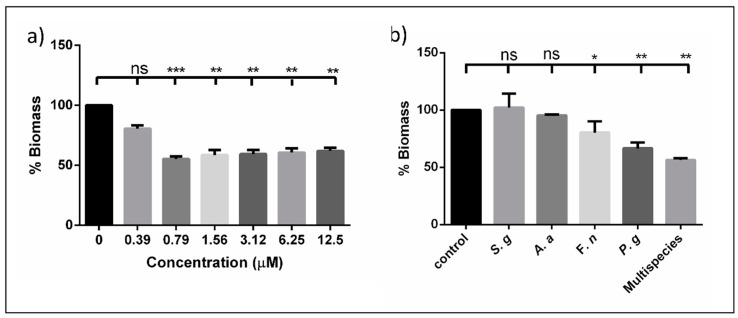
Effect of DMTU on biofilms. (**a**) Effect of sub-inhibitory concentrations of DMTU on multispecies biofilms shows significant reduction in biomass with BIC_50_ at 0.79 µM; (**b**) shows the effect of DMTU at BIC_50_ against mono-species and multispecies biofilms. Control was normalized to 100% and the significance was calculated. * denotes *p* ≤ 0.05, ** denotes *p* ≤ 0.01 and *** denotes *p* ≤ 0.001, ns denotes > 0.05; *S.g—S. gordonii, A.a—A. actinomycetemcomitans, F.n—F. nucleatum, P.g—P. gingivalis.*

**Figure 3:**
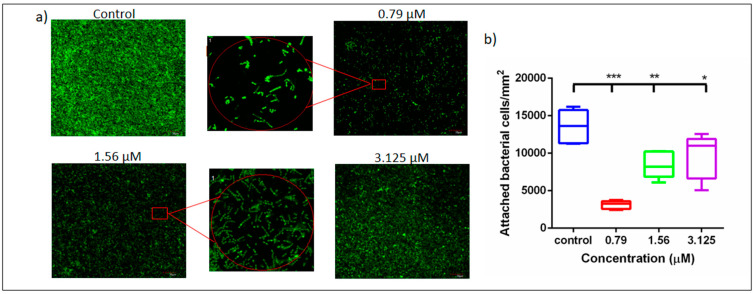
Confocal laser scanning images showing inhibition of multispecies biofilms by DMTU. (**a**) Panel shows the reduced green fluorescence (indicating less biofilm formation) when treated with different concentrations of DMTU; inset picture shows predominance of *S. gordonii* in treated biofilms; (**b**) total number of attached cells in each biofilm scaffold showing significant inhibition of biofilm formation at the tested concentrations. * denotes *p* ≤ 0.05, ** denotes *p* ≤ 0.01 and *** denotes *p* ≤ 0.001.

Concentrations ranging 12.5 µM (up to 0.79 µM) were able to inhibit multispecies biofilms significantly more effectively than the controls (*p* ≤ 0.05). The BIC_50_ (50% Biofilm Inhibitory Concentration) was identified at 0.79 µM (Figure 2). Notably, against mono-species biofilms, this concentration was able to inhibit only *P. gingivalis* (40% inhibition) and *F. nucleatum* biofilms (~25% inhibition), and it had no effect on *S. gordonii* or *A. actinomycetemcomitans* biofilms. Interestingly, DMTU was able to inhibit *P. gingivalis* biofilms but not *S. gordonii* in a range of concentrations (Figure S1). Such an effect has been reported for arginine, wherein it enhances the growth and biofilm formation of alkali-generating bacteria such as *S. gordonii* in multispecies biofilms, while preventing *P. gingivalis* biofilm formation [45]. Whether DMTU has similar mechanisms of action needs further research. These results were further confirmed by our confocal laser scanning microscopic (CLSM) analyses, wherein DMTU-coated substrates reduced bacterial adhesion and biofilm formation compared to the control (Figure 3a). At 0.79 and 1.56 µM, there was a significant reduction in the number of adherent cells/mm^2^ compared to the control (Figure 3b).

**Figure 4:**
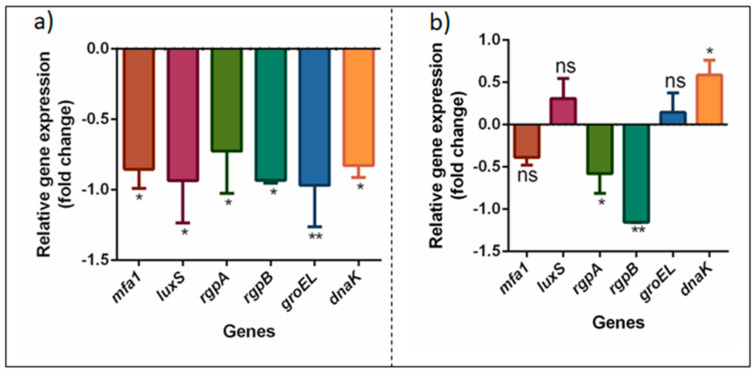
Differential gene expression in *P. gingivalis* following DMTU treatment of (**a**) multispecies and (**b**) mono-species biofilm. * denotes *p* ≤ 0.05, ** denotes *p* ≤ 0.01 and *** denotes *p* ≤ 0.001, ns denotes *p* > 0.05.

Based on these results, we asked if DMTU inhibited multispecies biofilms by inhibiting inter-species communication (quorum sensing) mechanisms. Therefore, we investigated the effects of DMTU on the biofilm- and virulence-related genes of *P. gingivalis* in mono-species and multispecies biofilms (Figure 4). The minor fimbriae of *P. gingivalis*, *mfa1*, interacts with dendritic cell receptors, and helps in its persistence by reducing the levels of pro-inflammatory cytokines. *mfa1* also binds to the cell wall receptor, SspA/B, of the early colonizer *S. gordonii* [46], thereby facilitating the adhesion of *P. gingivalis* to *S. gordonii*. This also initiates signal transduction events involving the tyrosine kinase dephosphorylation of the gene *ptk1*, which is essential in the synergistic interaction of *P. gingivalis* with other species [24]. Significant downregulation of these genes suggests that DMTU inhibits polymicrobial synergistic interactions.

It was previously shown through in silico studies that DMTU is a peptidase domain inhibitor of the ABC transporter protein (ComA) in *S. mutans* [33,34]. This study showed the targeted activity indirectly, by considering only the virulence genes downstream of the ComA protein and that the *comA* gene was not downregulated. On the other hand, recent studies have shown that different derivatives from 1,3-disubstituted urea possess anti-biofilm activity against *Pseudomonas aeruginosa*, *Mycobacterium tuberculosis* and *Candida albicans* [47]. These derivatives appear to specifically bind to the LasR protein, as reported by in silico docking studies [47]. Therefore, it is clear that 1,3-disubstituted urea derivatives can impart their biological activity against different species and kingdoms through a wide range of single or multitargeted interactions, which remain to be dissected in detail.

LuxS/AI-2 signaling is considered the universal communication system, and is widely conserved in a large number of Gram-negative and Gram-positive bacteria [48]. It serves multiple functions, including inter-species interactions, intraspecies regulatory mechanisms and host–microbe interactions [49]. In *P. gingivalis*, *luxS* is also involved in the activation of stress response genes, hemin, and iron acquisition genes [50,51]. It has been shown that AI-2 produced by *A. actinomycetemcomitans* and *S. gordonii* can complement *luxS* mutation in *P. gingivalis.* Furthermore, AI-2 produced by *F. nucleatum* initiates the activation of adhesion-related genes in *P. gingivalis* [25]. The results of this study clearly indicated that DMTU significantly downregulates *P. gingivalis luxS* in multispecies, but not in mono-species, biofilms (Figure 4). This may be due to the effect of the multispecies interaction with DMTU, which affects the *luxS* gene’s expression. The specific target(s) that downregulates the virulence genes of *P. gingivalis* in a multispecies biofilm are yet to be explored. Taken together, these findings corroborate our biofilm inhibition data, which showed the superior inhibitory effects of DMTU against multispecies biofilms compared to mono-species biofilms.

*P. gingivalis* is best known for its manipulation of the host immune system, which is solely controlled by the gingipain system [6]. Gingipains (*rgp*A, *rgp*B) are proteases which invade gingival epithelial cells by degrading E-cadherin (cell-cell junction) [52] and immunomodulate (by affecting the complement pathway activation) chemokine, cytokines and the degradation of antibodies, all of which collectively assists in its survival within the host system [6]. Gingipains are necessary for utilizing heme from hemoglobin in vivo and in vitro, making them essential for *P. gingivalis* growth, biofilm development and persistence in host cells [53,54]. Both the arginine-specific proteases (*rgp*A, *rgp*B) were downregulated significantly in mono-species and multispecies biofilms, indicating that DMTU was able to inhibit the virulence mechanisms of *P. gingivalis*.

In periodontal pockets, *P. gingivalis* is exposed to a variety of stress conditions, including temperature, pH and oxidative stress. In the diseased sites, the temperature is elevated relative to the healthy subgingival environments, thereby activating *dnaK* and *groEL* (heat shock proteins) to neutralize the stress [55]. DMTU significantly downregulated the *luxS*-regulated stress response pathways in multispecies biofilms, suggesting the inability of *P. gingivalis* to overcome temperature-mediated stress. Taken together, our phenotypic and gene expression data collectively demonstrate that DMTU is able to modulate *P. gingivalis* biofilm formation, virulence and stress response pathways.

### 3.2. DMTU Significantly Reduces Preformed Biofilm Biomass without Affecting Cell Viability

**Figure 5:**
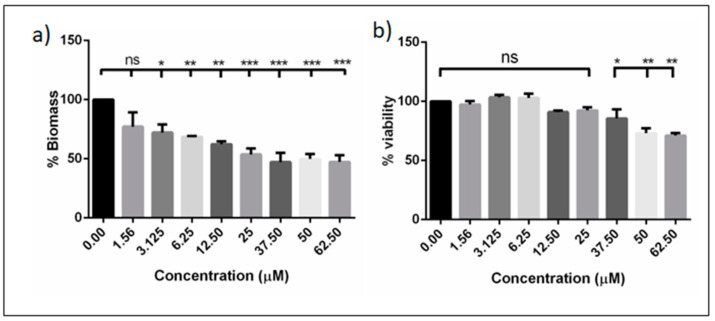
Effect of DMTU on preformed biofilms. (**a**) Dose-dependent reduction of biomass when treated with different concentrations of DMTU; (**b**) effect of DMTU on cell viability of pre-formed biofilms. * denotes *p* ≤ 0.05, ** denotes *p* ≤ 0.01 and *** denotes *p* ≤ 0.001, ns denotes > 0.05.

In clinical situations, a biofilm is rapidly formed in intraoral sites. Hence, we asked if the subMIC concentrations of DMTU could have potential effects on biofilm biomass without killing bacterial cells. Our results showed that the tested concentrations > 3.125 µM were able to significantly reduce biofilm biomass, compared to the control (*p* ≤ 0.05). Interestingly, these effects were dose-dependent (Figure 5). However, these concentrations of DMTU had no effect on the biomass of mono-species biofilms, except for that of *F. nucleatum* (Figure S2). The selective disruption of the biomass of the bridge colonizer *F. nucleatum* may explain the effects on multispecies biofilms. To confirm if the disruption of these multispecies biofilms was independent of bacterial killing, the cell viability was quantified using the XTT assay. No significant effects were observed on the cell viability up to 37.5 µM, whereas at 50 µM and 62.5 µM, a 20% reduction in cell viability was observed, when compared to control.

**Figure 6:**
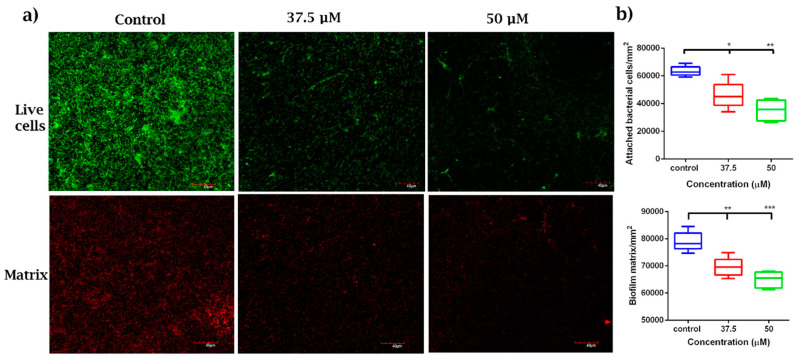
DMTU treatment of pre-formed biofilms. (**a**) Panel shows CLSM images of matrix (stained red by the SyPRO matrix stain) and live cells (stained green by Syto9) when treated with DMTU; (**b**) quantitative analyses of attached bacterial cells/mm2 and biofilm matrix/mm2 showed significant reduction in biofilm matrix by DMTU. * denotes *p* ≤ 0.05, ** denotes *p* ≤ 0.01 and *** denotes *p* ≤ 0.001.

Biofilm cells are embedded in an extracellular polymeric matrix, which forms a protective barrier that immobilizes the cells, enhancing interaction among the bacterial species, including cell–cell communication, the transfer of genetic material and the forming of a spatio-temporally organized biofilm consortia [56]. The matrix also prevents the diffusion of antimicrobial substances into the biofilm, making biofilms remarkably more tolerant to antimicrobials than their planktonic counterparts. We questioned if DMTU had any effect on the biofilm matrix. At concentrations of 37.5 µM and 50 µM, DMTU significantly reduced the biofilm matrix compared to the control (Figure 6). Syto9 stains both live and dead cells. However, since the biofilms were treated with sub-inhibitory concentrations of DMTU, here the Syto9 stained cells represent only the live cells. Therefore, the reduction in Syto9-stained cells may be attributed to the dissolution of the matrix, thereby washing away the loosely-bound live cells. Thus, the reduction in the protein components of the matrix following DMTU treatment suggests the disruption of the biofilm matrix when compared to control.

There are some limitations to this study. We did not assess the species-specific spatio-temporal changes in the DMTU-treated biofilms. Furthermore, we did not test the biofilm inhibitory effects under different environmental conditions. These will be established in future studies. In conclusion, our in vitro study highlights that DMTU has notable effects on multispecies biofilms, and downregulates genes related to inter-species communication and virulence.

## 4. Conclusions

The results of these comprehensive investigations demonstrate that the small molecule DMTU inhibits multi-species biofilm formation. Mechanistic studies determined that it downregulates a battery of virulence genes of the keystone periodontal pathogen *P. gingivalis* when present in polymicrobial communities, supporting the conclusion that it affects the synergistic alliance amongst the microbial communities. DMTU also effectively disrupts mature biofilms in a dose-dependent manner by affecting the biofilm matrix, thereby holding promise for its further develop as a prophylactic, as well as a therapeutic agent.

## Supplementary Materials

The following are available online at https://www.mdpi.com/2076-2607/8/9/1261/s1, Figure S1: Effect of DMTU on biofilm inhibtion of P. gingivalis and S. gordonii, Figure S2: Effect of DMTU on preformed biofilms of mono-species biofilms, Table S1: Primer sequences of P. gingivalis for qRTPCR analysis.
