# Supplementary material for: A Novel Small Molecule, 1,3-di-m-tolyl-urea, Inhibits and Disrupts Multispecies Oral Biofilms
Source: Microorganisms. 2020 Aug 20;8(9):1261. doi: 10.3390/microorganisms8091261 (PMC7570320; doi:10.3390/microorganisms8091261)
Supplement: Supplementary file 1 [file microorganisms-08-01261-s001.pdf]

**Supplementary file:**

**Table 1.** Primer sequences of *P. gingivalis* for qRTPCR analysis.

| Genes | Primer sequences | References |
|---|---|---|
| 1.*mfa-1* | F (5′ TTCCTACCAGCAGCACTTTCCA 3′)<br>R(5′TCGGCACTTGAGTTCCACTGATTAC3′) | [1] |
| 2.*luxS* | F(5′ GCAACTTATCTGCGTAATCATCC 3′)<br>R(5′ CCGTACCCGGCACTTCTCCTTC 3′) | [1] |
| 3.*rgpA* | F(5′ TGTTGGGAGGAATGGCGTT 3′)<br>R (5′ GTCGTACTTGTGGGTTGCGA 3′) | [2] |
| 4.*rgpB* | F(5′CCTACGTGTACGGACAGAGCTATA 3′)<br>R (5′-AGGATCGCTCAGCGTAGCATT 3′) | [3] |
| 5.*16srRNA* | F( 5′CATAGATATCACGAGGAACTCCGATT 3′)<br>R(5′AAACTGTTAGCAACTACCGATGTGG 3′) | [2] |
| 6.*groEL* | F(5′CGGCTACATCTCTCCCTACTTCGT 3′)<br>R(5′GAGGATCGGGAGCATCTCTTTCAG3′) | [4] |
| 7.*dnaK* | F(5′CTGACCGGTGAGGTAAAGGATGTC3′)<br>R(5′CTTCGTCGGGATAGTGGTATTGGC3′) | [4] |

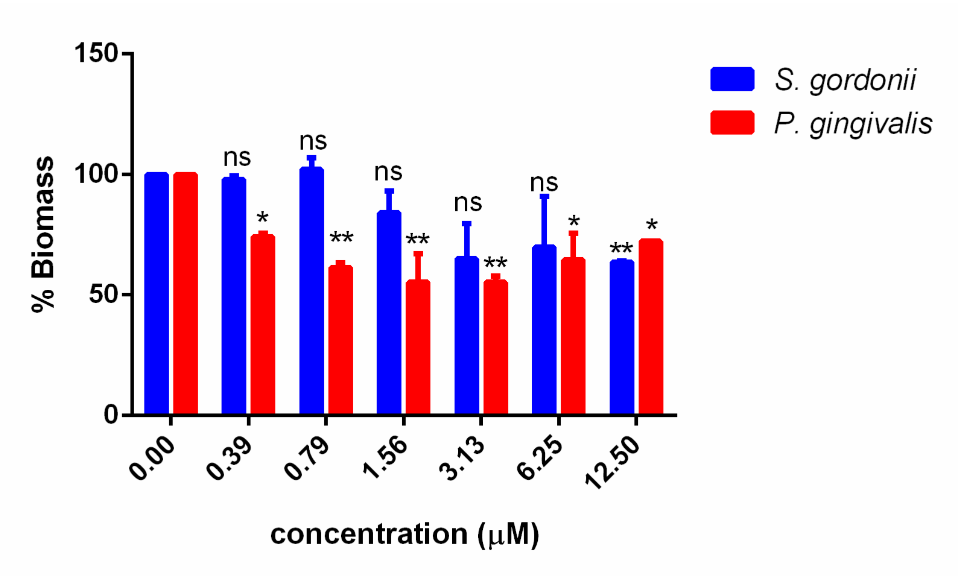


**Figure 1. Effect of DMTU on biofilm inhibtion of *P. gingivalis* and *S. gordonii*.** Significant reduction in biomass was observed in *P. gingivalis* in all concentrations tested, whereas in *S. goronii* signifucant reduction observed only in maximum concentration tested. . * denotes $p \leq 0.05$, ** denotes $p \leq 0.01$ and *** denotes $p \leq 0.001$, ns denotes not-significant.

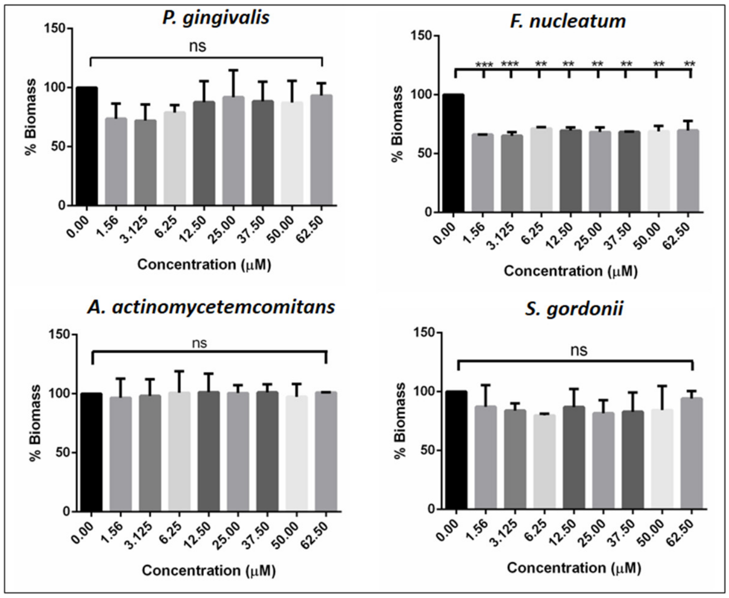


**Figure 2. Effect of DMTU on preformed biofilms of mono-species biofilms**. Significant Reduction in biomass was observed only in *F. nucleatum* when treated with varying concentrations of DMTU. ** denotes $p \leq 0.01$ and *** denotes $p \leq 0.001$.

## References:


[1] He, L.; Wang, H.; Zhang, R. and Li, H. The regulation of Porphyromonas gingivalis biofilm formation by ClpP. *Biochem. Biophys. Res. Commun.*, **2019**, *509*, 335–340.

[2] Shelburne, C.E.; Gleason, R.M.; Germaine, G.R.; Wolff, L.F.; Mullally, B.H.; Coulter, W.A.; Lopatin, D.E. Quantitative reverse transcription polymerase chain reaction analysis of Porphyromonas gingivalis gene expression in vivo. *J. Microbiol. Methods*, **2002**, *49*, 147–156.

[3] Saito, D.; Coutinho, L.L.; Saito, C.P.B.; Tsai, S.M.; Höfling, J.F.; Gonçalves, R.B. Real-time Polymerase Chain Reaction Quantification of Porphyromonas gingivalis and Tannerella forsythia in Primary Endodontic Infections. **2009**, *35*, 1518–1524.

[4] Hosogi, Y. and Duncan, M.J. Gene expression in Porphyromonas gingivalis after contact with human epithelial cells. *Infect. Immun.*, **2005**, *73*, 2327–2335.
